# PUM2 binds SARS-CoV-2 RNA and PUM1 mildly reduces viral RNA levels, but neither protein affects progeny virus production

**DOI:** 10.1099/jgv.0.002152

**Published:** 2025-09-16

**Authors:** Nhi Phan, Yelizaveta Zaytseva, Chia-Ching Lin, Mitali Mishra, Weina Sun, Paulina Pawlica

**Affiliations:** 1 Department of Microbiology, Icahn School of Medicine at Mount Sinai, New York, NY 10029, USA

**Keywords:** Pumilio, PUM1, PUM2, SARS-CoV-2

## Abstract

Pumilio proteins (PUM1 and PUM2) are essential post-transcriptional regulators of gene expression found across plants, animals and yeast. They bind Pumilio response elements (PREs) on messenger RNAs (mRNAs) to modulate mRNA stability and translation. PUMs have been implicated in diverse cellular processes, including stem cell maintenance, neurogenesis and cell cycle regulation. They have also been reported to negatively regulate innate immunity genes and to participate in viral RNA sensing. Previous high-throughput interactome studies revealed that PUMs bind severe acute respiratory syndrome coronavirus 2 (SARS-CoV-2) RNA. We found that SARS-CoV-2 transcripts contain multiple conserved PREs, some of which are preferentially bound by PUM2. Surprisingly, altering PUM levels does not affect the production of progeny virions. However, depletion of PUM1 slightly increases intracellular viral RNA levels, suggesting that PUM1 either plays a mild antiviral role against SARS-CoV-2 or regulates host factors that promote viral replication. Notably, PUM1 also negatively regulates innate immunity gene expression both at steady state and during SARS-CoV-2 infection. Our findings support a complex immunomodulatory role for PUM1, acting both as a negative regulator of innate immunity genes and a mild inhibitor of SARS-CoV-2 RNA accumulation. However, in cell culture, these roles appear negligible based on viral progeny output. Whether the multiple PREs found in the SARS-CoV-2 genome contribute to evasion of PUM1 activity remains an open question.

## Introduction

Pumilio 1 (PUM1) and Pumilio 2 (PUM2) are conserved, sequence-specific RNA-binding proteins that function as essential post-transcriptional regulators of gene expression, playing critical roles in growth, development, neurological processes and fertility [1,2]. Mutations in these proteins have been linked to human diseases, including ataxia and various cancers [1,2]. PUMs bind specific sequences known as Pumilio response elements (PREs) (consensus 5′-UGUAHAUA, where H = A, C or U) on messenger RNA (mRNAs) via their highly conserved C-terminal homology domain [3] to control mRNA stability and translation. Both PUM1 and PUM2 recognize the same PREs and largely share overlapping mRNA targets. However, PUM1 contains an additional N-terminal domain not present in PUM2, suggesting functional differences. Indeed, several studies have shown that the roles of PUM1 and PUM2 are not completely redundant [4,9].

In addition to their role in mRNA regulation, PUMs participate in stress responses, as evidenced by their translocation into stress granules under various stress conditions, including viral infections [10,11]. During infection, the innate immune system relies on pattern recognition receptors (PRRs), which detect pathogen-associated molecular patterns (PAMPs) to trigger immune response. Retinoic acid-inducible gene I (RIG-I)-like receptors (RLRs), including RIG-I and melanoma differentiation-associated protein 5 (MDA5), sense foreign RNA, leading the initiation of a signalling cascade that induces interferons, interferon-stimulated genes (ISGs) and pro-inflammatory cytokines [12].

Two studies examined the roles of PUM proteins in regulating innate immunity and viral replication, reporting opposite, likely virus-specific, effects on viral growth [8,10]. In 2014, Narita *et al*. reported that both PUMs act as positive regulators of RIG-I signalling by directly interacting with laboratory of genetics and physiology 2 (*LGP2*), another RLR, and enhancing its RNA-binding activity, thereby increasing the cell’s ability to detect foreign RNA [10]. The authors showed that depletion of PUMs increased, while their overexpression decreased, the RNA levels of Newcastle disease virus (NDV), a negative-sense paramyxovirus. Consequently, PUM depletion during NDV infection led to reduced induction of innate immunity genes, such as IFN-beta (*IFN-β*) and C-X-C motif chemokine ligand 10 (*CXCL10*). Importantly, this function was independent of the PUM RNA-binding domain, suggesting that it did not involve canonical regulation of mRNA targets. In contrast, a 2017 study by Liu *et al*. found that PUM1, but not PUM2, acts as a negative regulator of innate immunity signalling by repressing expression of *LGP2*, *CXCL10* and other immune genes [8]. This role was proposed to occur through post-transcriptional regulation of mRNA levels, especially that of *LGP2*. Notably, only about half of the genes upregulated upon PUM1 depletion were direct PUM1 targets, including *CXCL10* but not *LGP2*. Functionally, PUM1 depletion elevated basal expression of antiviral genes, which in turn suppressed replication of herpes simplex virus 1 (HSV-1), a double-stranded DNA virus. Thus, although both studies implicate PUMs in antiviral responses, they propose distinct mechanisms: one suggests a positive role for both PUMs in enhancing RIG-I signalling and antiviral activity, while the other describes PUM1 as a negative regulator of the antiviral state, implying a pro-viral role. These models are not mutually exclusive, as they employ different mechanisms; however, they indicate that the roles of PUMs in antiviral responses remain poorly understood and appear to be virus-specific.

In this study, we discovered that the severe acute respiratory syndrome coronavirus 2 (SARS-CoV-2) genome harbours conserved PREs and demonstrated that some of these elements are preferentially bound by PUM2. We confirmed that PUM1 is mildly antiviral against NDV. In the context of SARS-CoV-2 infection, we showed that PUM1 depletion slightly increases viral RNA levels but does not affect viral progeny output. We also observed that the loss of PUM1 leads to increased expression of innate immunity genes, including *IFN-β*, *IFIT2* and the known PUM target *CXCL10*. These findings suggest that PUM1 has a complex immunomodulatory role; however, at least in cell culture, SARS-CoV-2 neither exploits PUMs to enhance replication nor is affected by PUM1’s antiviral or immunomodulatory functions.

## Methods

### Cells

Calu-3 (ATCC) cells were cultured in EMEM (ATCC) and supplemented with 10% FBS (HyClone), penicillin-streptomycin-glutamine (Pen/Strep/Glut) (Gibco) and 1 mM sodium pyruvate (Gibco). A549-hACE2 (a kind gift from Adolfo García-Sastre) cells were cultured in DMEM (Gibco) with 10% FBS, Pen/Strep/Glut and 1 µg/ml blasticidin (Gibco). HEK293T (ATCC) and Vero E6 (ATCC) cells were cultured in DMEM (GIBCO) with 10% FBS and Pen/Strep/Glut.

### Generation of PUM knockout cell lines

PUM-targeting sequences (see Table 1) were cloned into lentiCRISPRv2 (Addgene, #49535). Lentiviral vectors were prepared in HEK293Ts by transfecting guide-containing lentiCRISPRv2 together with helper plasmids pMDG.2 and psPAX2 (Addgene, #12259, #12260). A549-hACE2 cells were transduced with lentiviral vectors at low multiplicity of infection (MOI), followed by puromycin (Gibco) treatment and clonal selection. Each clone was verified by Western blotting and Sanger sequencing of the gene loci. Two knockout clonal cell lines for each PUM were used.

**Table 1.** 

Sequence	Purpose
5′ CTTAAGGACTTACCGATGTG	PUM1 - targeting guide 1
5′ GAGCCAACACCGGATAACCT	PUM1 - targeting guide 2
5′ GTATACGGATCGGTCCCTGG	PUM2 - targeting guide 1
5′ TGATGAACCAAATCCACTGA	PUM2 - targeting guide 2
5′ CCTGTGCAAAGGAAATTAAGGAGA	qPCR for nsp2 F
5′ AGTCAGCACACAAAGCCAAAA	qPCR for nsp2 R
5′ TGTTGATGGTGTTCCATTTGTAGT	qPCR for RdRp F
5′ TCCTGATTATGTACAACACCTAGC	qPCR for RdRp R
5′ ACCTTAACTTGCCTGGTTGTG	qPCR for nsp14 F
5′ AAAAGCTGGTGTGTGGAATGC	qPCR for nsp14 R
5′ ACTTCAGAGTTTAGAAAATGTGGC	qPCR for nsp15 F
5′ ACTGGTACTTCACCCTGTTGT	qPCR for nsp15 R
5′ TGTCTCTGATGCAGATTCAACT	qPCR for nsp16 F
5′ AGTCTTAGGGTCGTACATATCACTA	qPCR for nsp16 R
5′ CAGTGCCTTTACTCCAGTGCC	qPCR for EVT4 F
5′ CTCAGGAAATTCCGTTGCTCT	qPCR for EVT4 R
5′ AGCGAAGTCCCGAACGACGA	qPCR for NORAD F
5′ TGGGCATTTCCAACGGGCCAA	qPCR for NORAD R
5′ ACATCATCCCTGCCTCTACT	qPCR for GAPDH F
5′ TCAGGTCCACCACTGACA	qPCR for GAPDH probe
5′ 5HEX-CAAGGTCAT-ZEN-CCCTGAGCTGAACGG-3IABkFQ	qPCR for GAPDH R
5′ GCTTCTCCACTACAGCTCTTTC	qPCR for IFN-β F
5′ 56-FAM-AGTGTCAGA-ZEN-AGCTCCTGTGGCAAT-3IABkFQ	qPCR for IFN-β probe
5′ CAGTATTCAAGCCTCCCATTCA	qPCR for IFN-β R
5′ GAGGCAGCGAACTCATCTTT	qPCR for ISG15 F
5′ 56-FAM/AGTACAGGA/ZEN/GCTTGTGCCGTGG/3IABkFQ	qPCR for ISG15 probe
5′ CCAGCATCTTCACCGTCAG	qPCR for ISG15 R
5′ CTTAGACATATTCTGAGCCTACAGC	qPCR for CXCL10 F
5′ CAGAATGGCAGTTTGATTCATGGT	qPCR for CXCL10 R
5′ CGGCTAACTCTGAGGACACG	qPCR for CDKN1b F
5′ AGAATCGTCGGTTGCAGGTC	qPCR for CDKN1b R
5′ ATCTTGGTCCCAGCTCTCCA	qPCR for TLK1 F
5′ GCTCATCCATTGCACCTTCC	qPCR for TLK1 R

### PUM double depletion and overexpression

For PUM double depletion, clonal cell lines derived from A549-hACE2, either controls (KO-ctl, 2 separate cell lines) or single knockouts of either PUM1 (clone 1 or 2) or PUM2 (clone 1 or 2), were additionally transfected with siRNAs (siCtrl, siPUM1 or siPUM2) for 48 h and then infected. Specifically, 2×10⁵ cells were seeded in a well of a 6-well plate and transfected the next day using RNAiMAX (Invitrogen) with a 10 nM mix of 2 siRNAs per gene (Silencer RNA, Ambion, #s18681, #s18680, #s23671, #s23673). For PUM overexpression, PUM reference sequences were cloned into pLVX-Puro (Takara) between the NotI and BamHI sites. Empty pLVX control or pLVX containing PUM constructs was transfected into HEK293Ts together with helper plasmids to generate lentiviral vectors. A549-hACE2 cells were seeded at 2×10⁵, transduced after 24 h with 1 ml of lentivirus-containing media and then infected after an additional 48 h. Successful depletion and overexpression were validated for each experiment.

### SARS-CoV-2 stocks and infections

SARS-CoV-2 stocks of isolate USA-WA1/2020 (BEI) were generated from passage 1 on VERO-E6 cells. Virus titres were determined by plaque assay as described previously [13]. A549-hACE2 cells were infected at an MOI of 0.5, and Calu-3 cells were infected at an MOI of 0.1. For IFN-alpha (IFN-*α*) (PBL assay science) pretreatment, cells were incubated overnight with 10³ U ml^−1^ and infected the next day. All infectious growth was performed in a biosafety level 3 laboratory and approved by the Mount Sinai Biosafety Committee.

### NDV stocks and infections

NDV stocks of the LaSota strain were generated and titrated as previously described [14,15]. Briefly, recombinant NDV was rescued using plasmids pNDV-LaSota, pTM1-NP, pTM1-P, pTM1-L and pCAGGs-T7opt in BSRT7 cells for 48 h. Afterwards, the homogenized cells and supernatant were injected into 9- to 11-day-old specific-pathogen-free embryonated chicken eggs (AvsBio), which were incubated at 37 °C for 2–3 days. Viruses in the allantoic fluids were purified through ultracentrifugation on a 20% sucrose cushion and titrated on Vero E6 cells. For infection, an MOI of 0.1 was used. Viruses were diluted in PBS containing 0.2% BSA (MP Biomedicals), Pen/Strep, calcium chloride and magnesium chloride. After a 1-h incubation, the inoculum was removed, and DMEM containing 0.3% BSA, Pen/Strep and 0.2 μg ml^−1^ tosylsulfonyl phenylalanyl chloromethyl ketone trypsin was added. Twenty-four hours post-infection (hpi), the supernatants were harvested, and viral titers were measured by plaque assay on Vero E6 cells.

### RNA immunoprecipitation

RNA immunoprecipitation (RIP) was performed as previously described [13]. Briefly, 5×10⁶ Calu-3 cells were infected with SARS-CoV-2 WA1 at an MOI of 0.05 for 48 h. Cells were lysed in 1 ml of IP Lysis Buffer (Pierce) in the presence of RNase inhibitor (NEB) and Protease Inhibitor Cocktail (Pierce). Lysates containing 400 µg of protein were incubated with 30 µl of Dynabeads Protein G (Invitrogen) coupled to 10 µg of antibody (anti-PUM1 or anti-PUM2, Bethyl) or with beads only as a no-antibody control. After IP, the beads were washed twice in Pierce Lysis Buffer and then five times in NET-2 buffer [50 mM Tris (pH 7.5), 150 mM NaCl and 0.05% Nonidet P-40]. Beads were resuspended in TRIzol (Invitrogen), and RNA was extracted and analysed by reverse transcriptase quantitative PCR (RT- qPCR).

### Reverse transcriptase qPCR

Total RNA was extracted from TRIzol according to the manufacturer’s recommendations. Two micrograms of RNA were subjected to DNase I digestion (Promega) and purified with acid phenol. One microgram of DNase-treated RNA was reverse transcribed using ProtoScript II and random hexamers. Quantitative PCR was performed using either SYBR Green qPCR Master Mix (Thermo Fisher) or Luna qPCR Master Mix (NEB). The reactions were performed according to the manufacturer’s instructions. Sequences are listed in Table 1.

### EdU incorporation

The cell proliferation assay was performed using the Click-iT EdU Proliferation Assay (Invitrogen) according to the manufacturer’s instructions. Briefly, 10^4^ cells from each PUM knockout clone, pretreated with siRNAs for 48 hours, were plated in a 96-well plate. The following day, the cells were incubated with 10 µM EdU for the indicated times. Afterwards, the cells were fixed, labelled with the Click-iT reagent, washed, blocked with 1.5% BSA and incubated with Amplex UltraRed for 15 min. The signal was measured using a Synergy H1m BioTek instrument.

### Western blot analysis

Cells were lysed in IP Lysis buffer (Pierce) containing protease inhibitor cocktail (Pierce). Approximately 20 µg of total protein was separated on a gradient SDS-PAGE gel (Invitrogen) and electrotransferred to a PVDF membrane (BioRad). After blocking with 5% milk in TBST [20 mM Tris (pH 7.5), 150 mM NaCl, 0.1% Tween 20], the membrane was probed with the appropriate antibodies and detected using ECL Plus Western Blotting Substrate (Pierce) on a ChemiDoc MP Imaging System. Primary antibodies used were anti-FLAG M2 (1:1000, F1804, Sigma-Aldrich), anti-PUM1 (1:2000, ab92545, Abcam or A300-201A, Bethyl), anti-PUM2 (1: 1000, A300-202A, Bethyl, or ab92390, Abcam) and anti-GAPDH (1:5000, G9545, Sigma-Aldrich). Secondary antibodies used included Sheep Anti-Rabbit IgG Antibody, HRP conjugate (1:5000, AP510P, Sigma-Aldrich) and Rabbit Anti-Mouse IgG Antibody, HRP conjugate (1:5000, AP160p, Sigma-Aldrich).

### Bioinformatic analysis

All-time per-nucleotide diversity statistics for SARS-CoV-2 (NC_045512.2) were downloaded from GISAID [13] on 9 July 2024. Missing values were set to zero. Rolling averages of diversity were calculated across 8-nt windows using a 1-nt step to smooth local variation, and the resulting values were *Z*-score normalized to identify regions of unusually high or low conservation. Diversity scores for PRE elements in the SARS-CoV-2 genome were then compared to those of all other 8-nt windows.

## Results

### PUMs bind conserved PREs on SARS-CoV-2 transcripts

**Fig. 1.**
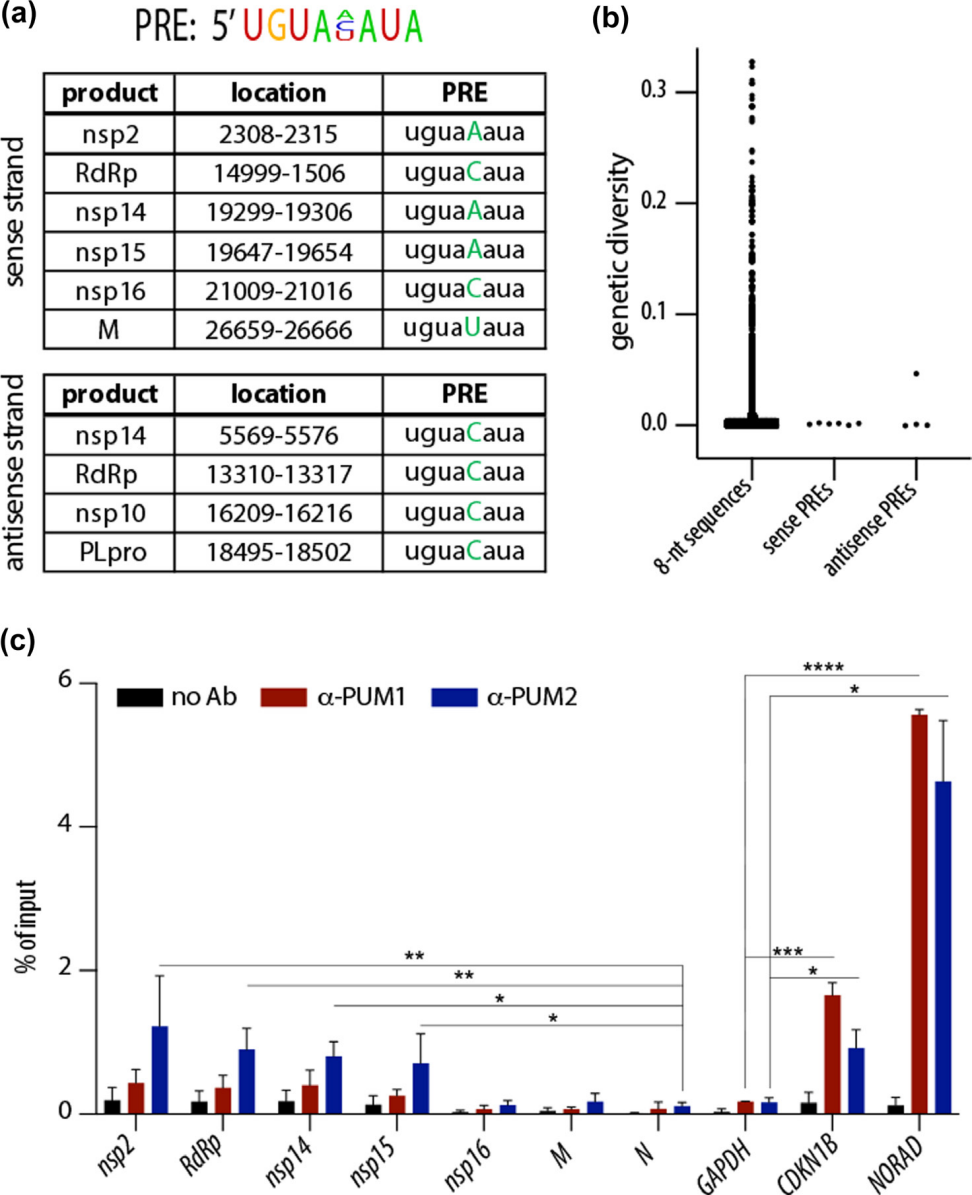
PUM2 binds conserved PREs on SARS-CoV-2 transcripts. (**a**) Top: Consensus motif of the PRE. Below: List of all PREs and their location in the SARS-CoV-2 genome. (**b**) Most of the PREs within the SARS-CoV-2 genome are genetically stable. Mutation rates for 8-nt sequences across the genome were calculated using diversity statistics from GISAID data. (**c**) SARS-CoV-2 transcripts are preferentially bound by PUM2. The graph shows mean values from RNA immunoprecipitations of PUM1 and PUM2 from Calu-3 cells infected with SARS-CoV-2 WA1 for 48 h at MOI 0.05 (*n*=3; error bars indicate sd). Significance was calculated using a ratio paired t-test by comparing values to the negative control: *GAPDH* for the host transcripts and *N* for the viral RNAs. * indicates a *P*-value of <0.05, **P<0.01, ***P<0.001 and ****P<0.0001. *CDKN1B* is a known PUM target, while *NORAD* contains ~20 PREs.

While analysing the SARS-CoV-2 genome, we noticed that it contains multiple PREs, with 6 located on the sense (genomic) strand and 4 on the negative-sense strand (Fig. 1a). Using GISAID’s [16] nucleotide mutation data for SARS-CoV-2, we calculated genetic diversity scores for each 8-nt sequence across the whole SARS-CoV-2 genome. Although the SARS-CoV-2 genome is generally stable, our analysis revealed that the PREs are highly conserved, with the exception of 1 located on the antisense strand that exhibits elevated genetic variability (Fig. 1b). Given that antisense transcripts are less abundant and likely confined to replication compartments, we focused primarily on PREs located on the sense strand. To determine whether these viral PREs are bound by PUM proteins, we performed RIP from lysates of SARS-CoV-2-infected Calu-3 cells. Interestingly, 4 PREs located on the sense strand were found to be preferentially bound by PUM2, as compared to the negative control, the *N* transcript, which lacks PREs (Fig. 1c). SARS-CoV-2 transcripts showed lower enrichment than the known host PUM target cyclin-dependent kinase inhibitor 1B (*CDKN1B*) [9,17] or the long non-coding RNA *NORAD*, which sequesters PUMs via ~20 PREs [18]. Some of the predicted PRE-containing viral transcripts were not bound by PUMs, which may be due to occlusion of binding sites by RNA structure or other RNA-binding proteins. Importantly, three independent high-throughput studies investigating protein interactions with SARS-CoV-2 transcripts identified PUM proteins as associated with viral RNA. Two of these studies detected PUM2 [19,20], and all three identified PUM1 [19,21]. Since both PUM1 and PUM2 recognize the same RNA motif (PRE), it is likely that PUM1 also binds SARS-CoV-2 RNA, even though we primarily detected PUM2. In summary, our data and those of others show that SARS-CoV-2 harbours conserved PREs, some of which are bound by PUM proteins, particularly PUM2.

### PUM1 depletion mildly increases SARS-CoV-2 RNA levels but does not affect progeny virus production

**Fig. 2.**
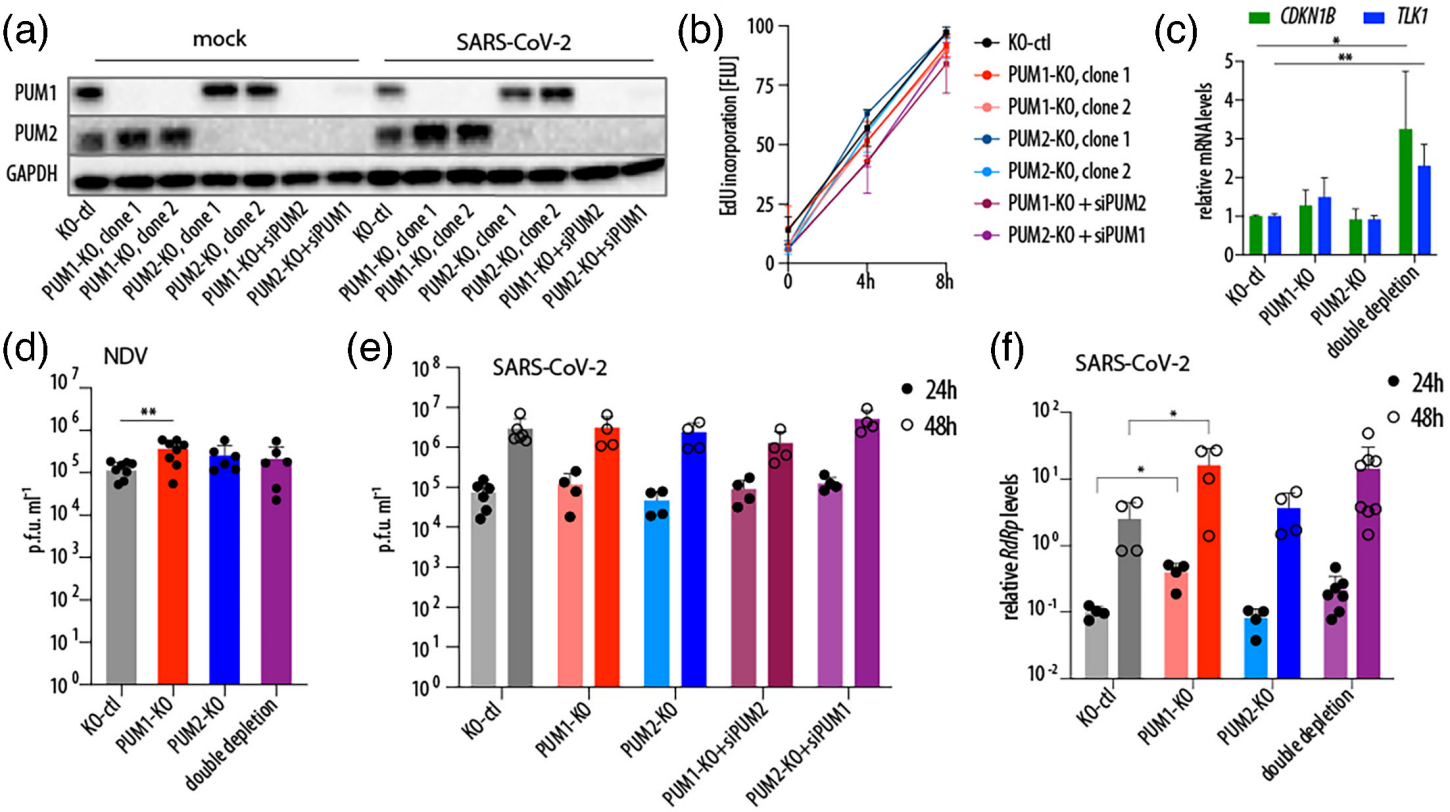
SARS-CoV-2 replication is largely unaffected by PUM depletion. (**a**) Validation of successful PUM depletion. A representative Western blot from uninfected and SARS-CoV-2-infected cells at 48 hpi. Clonal cell lines derived from A549-hACE2, either control (KO-ctl) or single knockouts (KO) of PUM1 or PUM2, were treated with siRNAs (siPUM1, siPUM2 or non-targeting control). 48 h post-siRNA treatment, cells were either infected or mock-infected with SARS-CoV-2 WA1 at an MOI of 0.5. (**b**) Cells with PUM depletion are viable. Viability was assessed by EdU incorporation into newly synthesized DNA. The data represent the average of two experiments performed in three biological replicates. (**c**) PUM double depletion de-represses known PUM targets, *CDKN1B* and *TLK1*. *P<0.05 and **P<0.01 (ratio paired t-test, *n*=3). (**d**) PUM depletion modestly increases NDV replication. A549-hACE2 cells, either controls or with single or double PUM depletion, were infected with the NDV LaSota strain at MOI 0.1. Viral replication was measured by plaque assay at 24 hpi. **P<0.01 (paired t-test, *n*=6). (**e**) SARS-CoV-2 progeny virus production is unaffected by PUM depletion. A549-hACE2 cells, either controls or with single or double PUM depletion, were infected with SARS-CoV-2 WA1 at an MOI of 0.5, and replication was measured by plaque assay. Graphs represent results from at least two experiments, each including two control cell lines, two clonal knockouts of each PUM and four double depletion conditions. (**f**) SARS-CoV-2 RNA levels are slightly increased by PUM1 depletion. The experiment was set up as in (**e**), and intracellular viral RNA levels were measured by RT-qPCR and normalized to *GAPDH*. *P<0.05 (paired t-test). Error bars indicate sd. EdU, 5-ethynyl-2′-deoxyuridine; RdRp, RNA-dependent RNA polymerase.

Since PUM proteins have been shown to modulate innate immunity, and both our data and previous studies have detected their binding to the SARS-CoV-2 genome, we reasoned that the virus may either exploit PUMs to promote its replication or sequester them to inhibit their antiviral function. To assess the impact of PUM depletion on SARS-CoV-2 replication, we used CRISPR-Cas9 to generate single PUM knockouts in A549-hACE2 cells, with two independent clones for each protein. Because cells with double PUM knockout exhibit growth delays due to prolonged de-repression of PUM targets [19,20], we employed a strategy in which one PUM was knocked out and the other was depleted using siRNAs (Fig. 2a). We measured cell viability via DNA synthesis and confirmed that cells continued to proliferate despite transient double PUM depletion (Fig. 2b). We further validated our system by confirming that depletion of both PUMs leads to de-repression of their known targets: *CDKN1B* and Tousled-like kinase 1 (*TLK1*) [9,17, 22] (Fig. 2c). Since Narita *et al*. reported that PUMs have antiviral activity against NDV [10], we asked whether this observation is reproducible in A549 cells. We confirmed that PUM1, but not PUM2, negatively regulates NDV replication, as measured by viral titers (Fig. 2d). In the aforementioned study, NDV replication was measured by viral RNA levels – a more sensitive method capable of detecting subtle effects, such as those potentially mediated by PUM2. Surprisingly, in the context of SARS-CoV-2 infection, neither single nor double PUM depletion affected viral replication, as measured by progeny virion production (Fig. 2e). Interestingly, viral RNA levels were slightly upregulated in PUM1-depleted cells, but not in PUM2-depleted cells, suggesting that PUM1 may have a mild antiviral role against SARS-CoV-2 or may regulate a host factor that promotes viral replication (Fig. 2f). If PUM1 is indeed antiviral, the lack of major activity against SARS-CoV-2 might indicate that the virus has evolved a strategy to evade this function. In summary, we confirm a mild antiviral activity of PUM1 against NDV and observe a subtle inhibitory effect on SARS-CoV-2 RNA levels; however, depletion of PUMs have no significant impact on progeny virus production.

### PUM overexpression does not affect SARS-CoV-2 progeny virus production

**Fig. 3.**
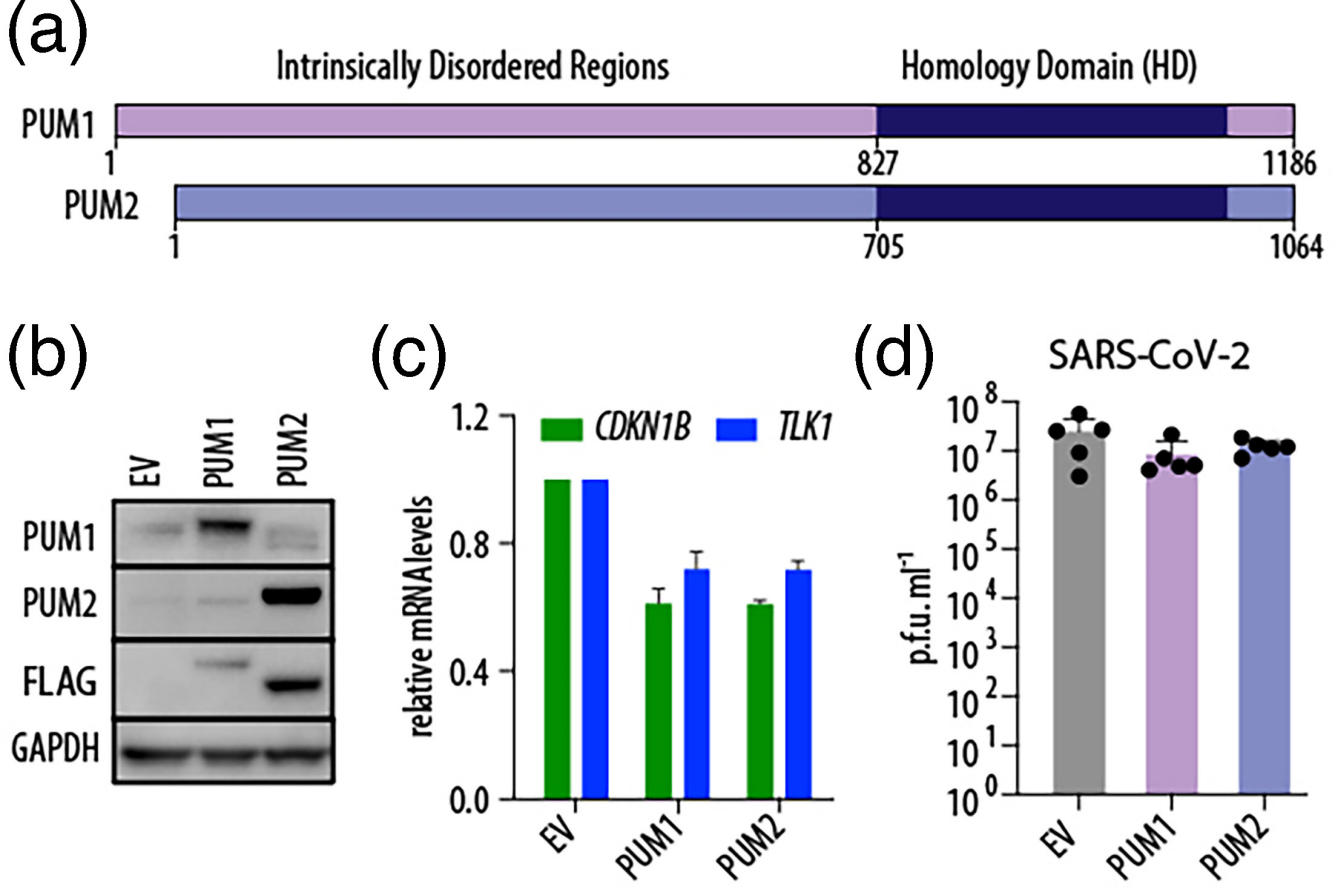
PUM overexpression does not affect SARS-CoV-2 replication. (**a**) Schematic representation of PUM proteins. (**b**) Representative Western blot from A549-hACE2 cells transiently transduced with the indicated FLAG-tagged constructs. (**c**) PUM overexpression represses known PUM targets, *CDKN1B* and *TLK1* (*n*=2). (**d**) PUM overexpression does not impact SARS-CoV-2 replication. A549-hACE2 cells were transduced with the indicated constructs and infected with SARS-CoV-2 WA1 at MOI 0.5 for 48 h. Viral replication was measured by plaque assay (*n*=5). Error bars represent sd.

We also investigated whether PUM overexpression affects SARS-CoV-2 replication. A549 cells are notoriously difficult to transfect, and we observed that stable transduction leads to rapid loss of exogenous PUM expression, possibly due to silencing by the HUSH complex [23]. Additionally, PUM protein levels are tightly regulated through mechanisms including auto- and cross-regulation via PREs within their own mRNAs [1,24], as well as sequestration by the long non-coding RNA *NORAD* [25,27]. Thus, to assess the effect of PUM overexpression, we transiently transduced A549-hACE2 cells with lentiviral vectors carrying PUM constructs (Fig. 3a, b). We first confirmed successful overexpression by demonstrating downregulation of known PUM targets, *CDKN1B* and *TLK1*. In the context of SARS-CoV-2 infection, PUM overexpression did not substantially affect viral titres (Fig. 3c). In summary, our data show that overexpression of PUM proteins plays a negligible role in the production of SARS-CoV-2 progeny virions.

### PUM1 negatively regulates innate immunity genes, but this activity is not enhanced by type I IFN treatment

**Fig. 4.**
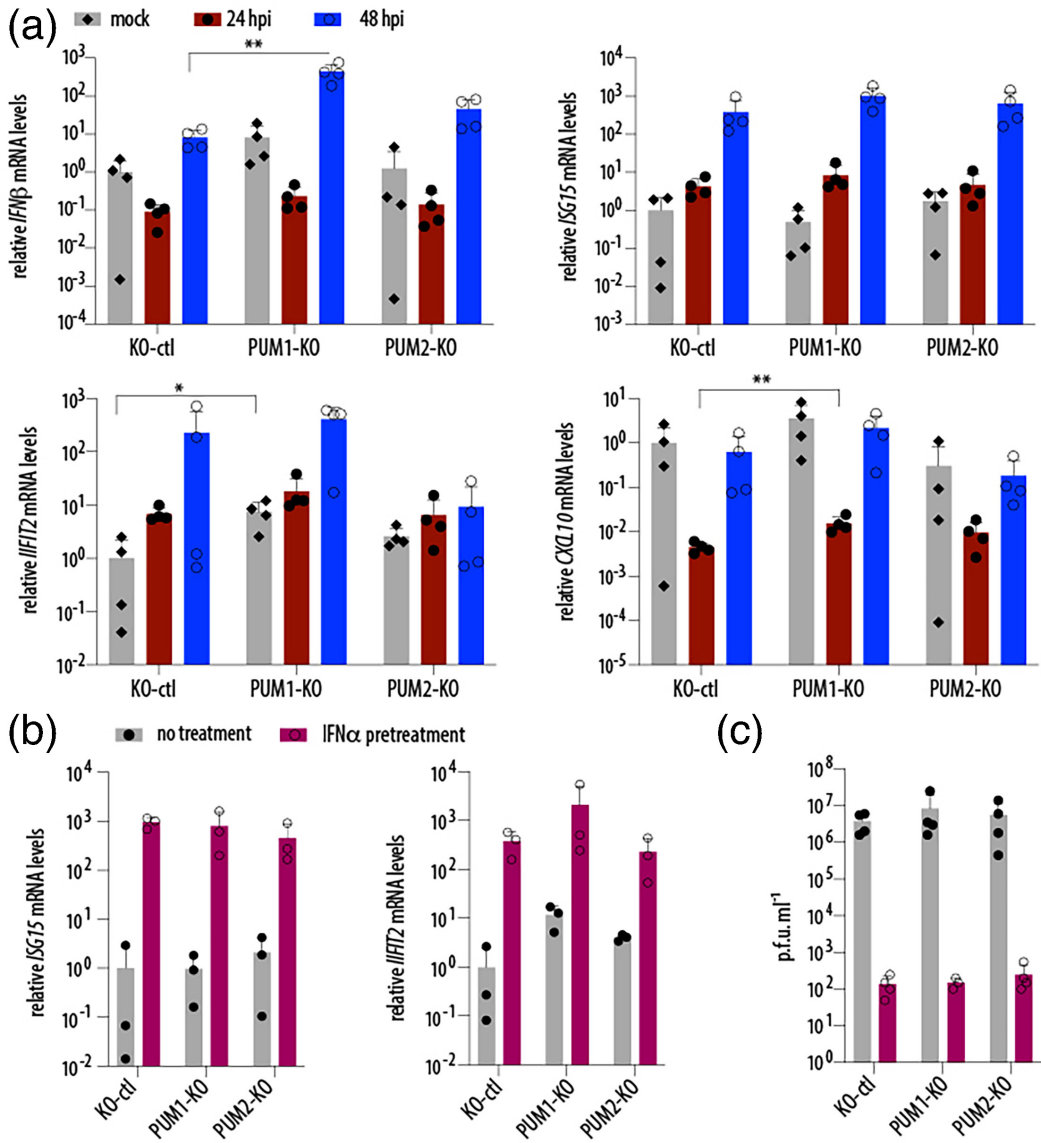
PUM1 negatively regulates innate immunity genes, but this activity is not enhanced by type I IFN pre-treatment. (**a**) PUM1 depletion negatively regulates expression of innate immunity genes during steady state and SARS-CoV-2 infection. A549-hACE2 cells, either control or with single PUM depletions, were infected with SARS-CoV-2 WA1 at an MOI of 0.5, and mRNA levels of *CXCL10*, *IFN-β*, *ISG15* and *IFIT2* were measured by RT-qPCR. Results were normalized to *GAPDH* and then to mock. *P<0.05, **P<0.01 (paired t-test, *n*=4). (**b**) PUM1 depletion does not enhance the antiviral state induced by IFN-*α* pretreatment. A549-hACE2 cells, either control or with single PUM depletions, were treated with 10³ U ml^−1^ of IFN-*α* for 24 h, after which RT-qPCR was performed (*n*=3). (**c**) Single PUM depletion does not affect the impact of IFN-*α* pretreatment on SARS-CoV-2 replication. A549-hACE2 cells, either control or with single PUM depletions, were pretreated overnight with 10³ U ml^−1^ of IFN-*α*and infected at an MOI of 0.5 with SARS-CoV-2 WA1. Viral replication was assayed 48 hpi. Error bars represent sd. RdRp, RNA-dependent RNA polymerase.

Two previous studies reported that PUM proteins regulate innate immunity genes during NDV and HSV-1 infections; however, the directionality of this regulation differed between the studies [8,10]. We thus investigated how PUM depletion affects the levels of *CXCL10*, *IFN-β*, *ISG15* and *IFIT2* at steady state and during SARS-CoV-2 infection (Fig. 4a). At steady-state levels (mock), the absence of PUM1 led to higher levels of most tested genes, except *ISG15*; however, statistical significance was reached only for *IFIT2*. During SARS-CoV-2 infection, induction of the antiviral programme was largely inhibited by the virus, consistent with its known ability to suppress IFN signalling [28,32]. There was a general trend that, in the absence of PUM1, innate immunity genes were expressed at higher levels compared to control cells. The genes that reached statistical significance at any time point during infection were *IFN-β* and *CXCL10*. This is in agreement with findings by Liu *et al*., who proposed that PUM1 acts as a negative regulator of innate immunity genes [8]. We then tested whether PUM1 depletion alters the antiviral state induced by IFN-*α* pretreatment and found no change (Fig. 4b). Consistently, the inhibitory effect of IFN-*α* pretreatment against SARS-CoV-2 [33,34] was unaltered by PUM1 depletion. Overall, these results suggest that although PUM1 negatively regulates the expression of innate immunity genes, these effects are negligible for SARS-CoV-2 replication in cell culture.

## Discussion

In this study, we identified genetically stable PREs present in the SARS-CoV-2 genome and showed that some of them are preferentially bound by PUM2 (Fig. 1c). Interestingly, other high-throughput studies have identified both PUM proteins as being associated with SARS-CoV-2 transcripts [19,21]. PUM1 and PUM2 are known to interact with each other [22], which may explain why the presence of one protein is often accompanied by the other. Also, since both PUM1 and PUM2 recognize the same RNA motif (PRE), it is likely that both bind to SARS-CoV-2 RNA, although one may exhibit a preference depending on the surrounding RNA context. On the other hand, the selective binding of PUM2 might be biologically relevant, as in our experiments this bias is unlikely to result from technical issues such as antibody quality – both anti-PUM1 and anti-PUM2 antibodies efficiently pulled down host transcripts, with anti-PUM1 performing even better (Fig. 1c). One hypothesis for the preferential binding of PUM2 is that it may serve to shield the viral genome from PUM1’s antiviral properties.

Here, we confirmed that PUM1 is modestly antiviral against NDV (Fig. 2d). Although we did not observe major antiviral activity of PUM1 against SARS-CoV-2, as measured by progeny virion production, we detected slightly higher levels of intracellular viral RNA in the absence of PUM1, which could suggest the presence of residual antiviral function (Fig. 2e, f). It is unclear whether the mechanism underlying these antiviral properties is the same as for NDV, as proposed by Narita *et al*. [10] PUMs would participate in foreign RNA sensing and thereby enhance downstream signalling. In contrast, we observed that PUM1 negatively regulates innate immunity, as proposed by Liu *et al*. [8] (Fig. 4a). Thus, our results are partially consistent with the two previously published studies and suggest that PUM1 has at least two distinct functions: one antiviral and one involving negative regulation of innate immunity genes. It is also possible that PUM1 is not directly antiviral, but instead regulates the expression of a host factor that promotes viral replication. However, in the context of SARS-CoV-2, the regulatory roles of PUM1 appear to be negligible, at least in cell culture. A limitation of this study is that all experiments were conducted in cell culture, and it remains unclear whether PUM1 plays a significant role in viral infection within an organism.

Our study expands the understanding of the immunomodulatory roles of PUM1 in the context of viral infection, specifically during SARS-CoV-2 replication. There is an intriguing discrepancy regarding the role of PUM1 in viral infections: it appears antiviral against NDV, proviral for HSV-1 and largely neutral for SARS-CoV-2. Two possible explanations may account for this. First, these viruses belong to distinct families and produce different PAMPs, which are recognized by different PRRs. This could influence PUM1’s involvement in foreign RNA sensing, as proposed by Narita *et al*. [10]. Second, given the antiviral function of PUM1 against NDV – a virus whose genome lacks PREs – it is tempting to speculate that SARS-CoV-2 and HSV-1, both of which contain multiple PREs, may use these elements to evade PUM1’s antiviral function. Mechanistically, PRE-mediated evasion could occur through preferential binding of PUM2, shielding the viral genome from PUM1’s effects, or through sequestration of PUM1, preventing its interaction with LGP2 and impairing its role in RNA sensing. These hypotheses, however, are difficult to test, as they would require introducing multiple mutations into viral genomes. At present, it remains unclear whether PUM1 has major antiviral activity against viruses, as even its activity against NDV appears modest at best.

## Abbreviations

*CDKN1B*: cyclin-dependent kinase inhibitor 1B
*CXCL10*: C-X-C motif chemokine ligand 10
hpi: hours post-infection
HSV-1: herpes simplex virus 1
IFN-*α*: IFN-alpha
IFN-*β*: IFN-beta
ISGs: interferon-stimulated genes
LGP2: laboratory of genetics and physiology 2
NDV: Newcastle disease virus
PAMPs: pathogen-associated molecular patterns
Pen/Strep/Glut: penicillin-streptomycin-glutamine
PREs: Pumilio response elements
PRRs: pattern recognition receptors
PUM1: Pumilio 1
PUM2: Pumilio 2
qPCR: quantitative PCR
RIG-I: retinoic acid-inducible gene I
RIP: RNA immunoprecipitation
RLRs: retinoic acid-inducible gene I-like receptors
*TLK1*: Tousled-like kinase 1
